# Is Month of Birth a Risk Factor for Colorectal Cancer?

**DOI:** 10.1155/2017/5423765

**Published:** 2017-01-04

**Authors:** N. K. Francis, N. J. Curtis, E. Noble, M. Cortina-Borja, E. Salib

**Affiliations:** ^1^Department of General Surgery, Yeovil District Hospital NHS Foundation Trust, Higher Kingston, Yeovil BA21 4AT, UK; ^2^Faculty of Science, University of Bath, Wessex House 3.22, Bath BA2 7AY, UK; ^3^Department of Surgery and Cancer, Imperial College London, Floor 10, QEQM Building, St. Mary's Hospital, Praed Street, London W2 1NY, UK; ^4^Centre for Paediatric Epidemiology and Biostatistics, Institute of Child Health, University College London, 30 Guilford St, London WC1N 1EH, UK; ^5^Faculty of Health and Life Sciences, University of Liverpool, Brownlow Hill, Liverpool L69 3BX, UK

## Abstract

*Introduction*. The developmental origins of health and disease hypothesis and season of birth have been linked to a wide variety of later life conditions including cancer. Whether any relationship between month and season of birth and colorectal cancer exists is unknown.* Methods*. A case-control study was performed with month of birth extracted from a dedicated colorectal cancer database. Age and gender matched patients were used as a control group. Generalised linear models were fitted with Poisson and negative binomial responses and logarithmic links. A forward stepwise approach was followed adding seasonal components with 6- and 12-month periods.* Results*. 1019 colorectal cancer patients and 1277 randomly selected age and gender matched controls were included. For both men and women there is an excess of colorectal cancer in those born in autumn and a corresponding reduction of risk among those born in spring (*p* = 0.026). For the identified September peak, the excess risk for colorectal cancer was 14.8% (95% CI 5.6–32.3%) larger than the spring trough.* Conclusion*. There is a seasonal effect in the monthly birth rates of people who are operated for colorectal cancer with a disproportionate excess of cancer in those born in September. Further large studies are required to validate these findings.

## 1. Introduction

The developmental origins of health and disease hypothesis states that intrauterine and early life environmental conditions have a significant impact upon health and development that persists through into adult life [1]. This concept was developed from the Barker hypothesis following the epidemiological identification of poor foetal and early life nutrition to an increased risk of coronary heart disease [2]. This paradigm shift altered our understanding to seeing later life risk as representing a combination of genetic predisposition, lifestyle factors, and early life events during the plastic phase of development.

Season or month of birth can result in differing early environmental exposure to a variety of stimuli such as sunlight, temperature, and infectious pathogens which could influence physiological and immunological development. Epidemiological studies have linked season of birth with a wide variety of conditions including lifespan and mortality risk [3–5], cardiovascular events [2, 6], type II diabetes [7], hypertension [8], mental health conditions [9], and suicide risk [10]. An association with season of birth has also been identified in malignant conditions such as childhood and adolescent haematological [11–13] and central nervous system cancers [14].

Proof of principle that season of birth can influence malignancy risk through long latency periods to adult onset cancer is suggested by available reports on skin [15, 16], breast [17], ovarian [18], and brain tumours [19]. There is a paucity of studies investigating gastrointestinal conditions [20, 21], particularly malignancies that typically present in later life.

The association between month of birth and the risk of developing colorectal cancer (CRC) has not been explored. Therefore, we aimed to investigate if there is a disproportional seasonality phenomenon in monthly birth rates of individuals who later develop CRC.

## 2. Methods

A case-control study using an observational review of a dedicated, prospectively populated database of patients undergoing surgery for CRC at Yeovil District Hospital, UK, was performed. All data was prospectively collected and managed by a dedicated information analyst team. Study inclusion criteria were surgical resection, with curative intent, for histopathologically proven colorectal adenocarcinoma after resection between 2002 and 2015. All eligible patients were included with month of birth extracted. All patients entered a standard clinical, radiological, and endoscopic follow-up until five years after surgery or death. A control group of inpatients, with age and gender matching, managed at the same hospital over the same time period with any other diagnosis than colorectal cancer was selected at random from UK NHS Hospital Episode Statistics data [22]. Control: case ratio was 1.25 to 1. Institutional approval for the creation of the database and review of already held anonymous data was obtained from the research ethics board at Yeovil District Hospital NHS Foundation Trust, UK. This report was prepared in keeping with the STROBE guidelines [23].

### 2.1. Statistical and Data Analysis

We fitted generalised linear models (GLM) with Poisson and negative binomial responses and logarithmic links. Overdispersion was formally assessed with a likelihood ratio test comparing Poisson and negative binomial models taking into consideration the problems of testing at boundary values. Trends by year of birth were adjusted using cubic splines with 3 effective degrees of freedom; this functional form was deemed complex enough to model these trends by comparing other possible spline models using Akaike's information criterion (AIC). Seasonal variation was modelled using at most three harmonic components in order to have enough flexibility to detect asymmetric seasonal variations which would be lost if only one symmetric, 12-month cycle had been fitted. The seasonal components used had 12 and 6 months; the first accounts for symmetric, yearly cycles, and the second was included in order to allow detecting semestral and quarterly patterns. The final models presented were selected by minimising the AIC and, as in all stepwise-type model selection procedures, correlation among the parameter estimates may produce misleading results—for instance entered variables can be proxies for others. The final models have a large number of parameters, and some of the effects, which appear to be significant when comparing the parameter estimates to their standard errors, may be spurious. All calculations were carried out using R version 2.8. The negative binomial GLM were fitted for male and female cases and controls using the MASS R-library. A* p* value of <0.1 was considered significant.

## 3. Results

2296 patients were included in this study. 1019 CRC patients (median age 73, range 35–96) met inclusion criteria with 1277 randomly selected age matched controls (Table 2).

**Figure 1:**
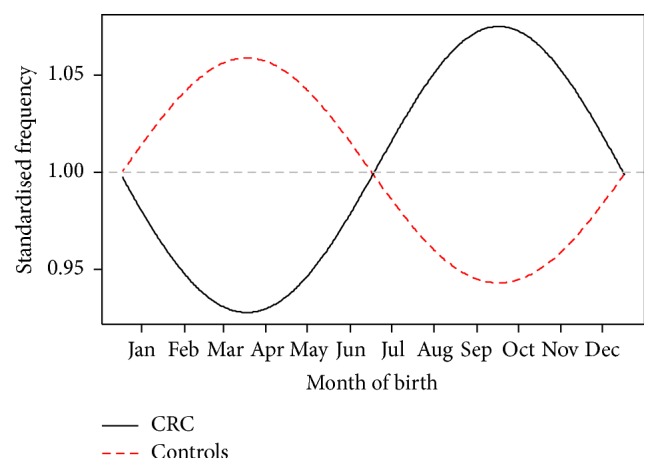
The smoothed graph shows the standardized predicted values by control/CRC status standardized by their means. There seems to be an excess of CRC cases born in early autumn and a corresponding reduction of risk among those born in spring. The seasonal birth patterns among CRC differ from those who did not have CRC (*p* = 0.026).

Figure 1 illustrates the fitted Poisson regression model to the 31-day month-adjusted frequencies of controls and CRC patients as a function of month of birth expressed as harmonic functions (sine and cosine) with periods 1 year and 6 months.  Table 1 demonstrates the interactions between these terms and status and showed that only the sine curve with period of 1 year and its interaction with status were significant. There is an excess of CRC cases born in early autumn and a corresponding reduction of risk among those born in spring (*p* = 0.026). For the peak at end of September, the excess risk for CRC was 14.8% (95% CI 5.6–32.3%) larger than that at the trough in spring.

**Figure 2:**
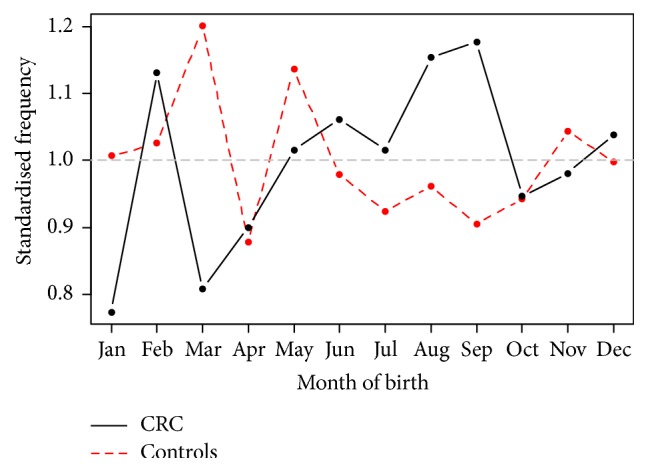
Detrended monthly seasonal components as a fraction of their mean for CRC. The trough (March) and the peak (September) of the seasonal component are similar to the Poisson model at 14% (*p* = 0.06).

**Figure 3:**
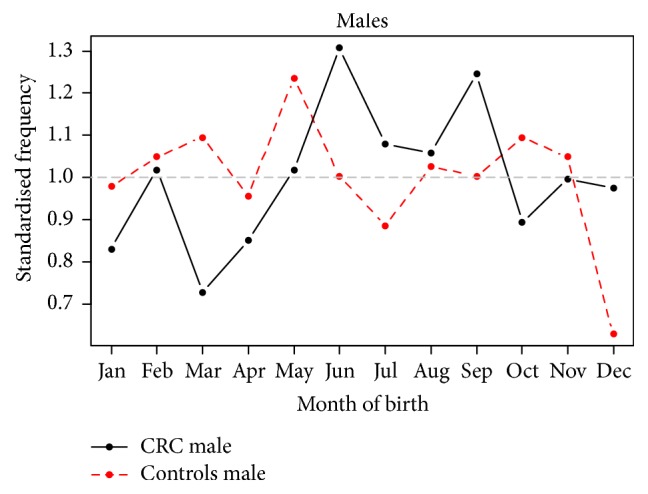
Detrended seasonal components for male CRC and controls as a fraction of their means. The average increase of risk between the spring and the autumn peak of the detrended seasonal components was 13% for men. In men, the spring trough remains but two peaks are noted in September and another one in June.

**Figure 4:**
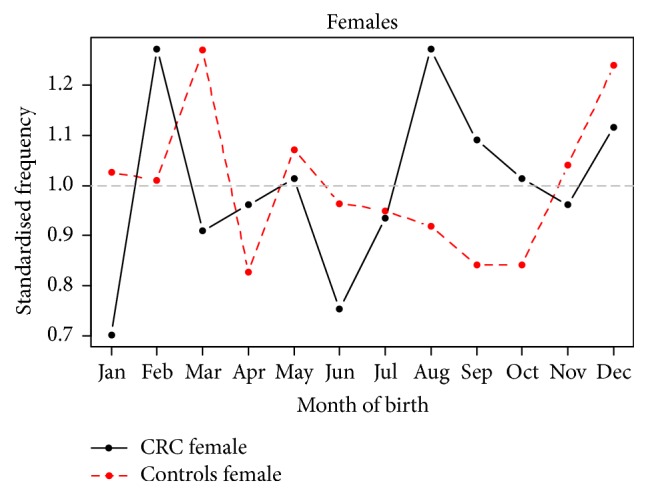
Detrended seasonal components by gender for female CRC and controls as a fraction of their means. An average increase of risk between the spring and the autumn peak of 16% for women is seen. The September peak remains in evidence but the trough appears to have shifted from March to January.

Generalised linear models for negative binomial responses with a logarithmic link function were fitted since there was significant overdispersion in the Poisson models. A forward stepwise approach was followed adding seasonal components with 12-month and 6-month periods, as well as interactions among them, the secular trend.  Figure 2 shows the detrended monthly seasonal components as a fraction of their mean for CRC. Adjusting for the year of birth, the model predicts that the average increase in risk of CRC; the trough (March) and the peak (September) of the seasonal component are similar to the Poisson model at 14% (95% CI 4.9–34%,* p* = 0.06). The February peak in CRC birth which was not present in the Poisson model may have resulted from adjusting for overdispersion in the negative binomial model. Figures 3 and 4 show the detrended seasonal components by gender for male and female CRC and controls as a fraction of their means, respectively, using the fitted negative binomial models for male and female cases and controls. The average increase of risk between the spring and the autumn peak of the detrended seasonal components was 16% (95% CI 12–21.8%) for women and an average of 13% (95% CI 5.2–22.7%) for men. The September peak remains in evidence in female patients but the trough appears to have shifted from March to January. For CRC male births, the spring trough remains but two peaks are noted in September and another one in June.

## 4. Discussion

We aimed to investigate any potential association between month of birth and undergoing surgery for colorectal cancer and observed a higher incidence among those born in the autumn months, particularly September, and a trough for those born in spring. This model supports the hypothesis of different seasonal phenomena by month of birth for both men and women who develop CRC. The higher birth rates in the autumn for people who later develop CRC suggest that seasonality of birth for CRC may exist. To our knowledge, this is the first report exploring this relationship and our findings need to be validated using larger datasets.

The mechanism behind month and CRC risk is unknown and correlation should not be interpreted as a causative association. It is unclear how early life events could contribute to CRC given the very long latency which greatly exceeds that of the reports investigating cancer in children and adolescents. The list of potential candidate exposures is vast and likely to be multifactorial including infections, many of which vary seasonally, maternal diet, environmental toxins, sunlight, and the hormonal milieu. On the basis of the foetal origins hypothesis, it may be plausible to suggest that the “month of birth” factor in CRC may reflect the timing of an errant early cellular changes or differentiation process due to one or more of these exposures, resulting in epigenetic programming [24] or chronic inflammation [25] leading to cellular abnormalities altering the individual's predisposition. This could act as a contributing, latent risk factor that might enhance other carcinogenic risk factors in predisposed individuals as the majority of nonmodifiable CRC risk factors are related to lifestyle. This assumption gives some hope that developmental approaches might improve our understanding of the pathogenesis and could offer new strategies for prevention. Should month of birth be confirmed as a risk factor for CRC, it is currently unclear if this would represent a nonmodifiable factor or one that a potential public health intervention may alleviate.

This exploratory study is subject to a number of limitations. It is impossible to rule out entirely the effect of random fluctuations in birth rates of people who later develop CRC or other confounding influences that might explain the findings. Our results may have been influenced by including only those patients that were amenable and fit to receive curative surgery. In order to be absolutely sure about the diagnosis of colorectal cancer, only patients with positive tissue diagnosis after surgery were selected to remove the possibility of cohort heterogeneity. Our strict inclusion criteria allowed us to be certain about our case group and ensure the cohort was homogenous. Whilst our data on this group was complete, future large scale validation studies should incorporate all diagnoses of CRC in order to truly investigate if month of birth is a risk factor. Meaningful subgroup analysis by age groups or site of colorectal cancer was not possible due to low numbers.

This study took place at one site and may not be generalisable to other geographical areas or populations particularly as patient ethnicity, place of birth, and time living in the UK were not included in this dataset. Seasonal environmental factors present in the childhood of included patients may not be applicable to children born today. Without previous data, it was not possible to perform a power calculation or calculate an effect size for the findings observed; therefore the inferential value of the study is undetermined.

## 5. Conclusion

Our results support the hypotheses that there is a seasonal effect in the monthly birth rates of people who later undergo colorectal cancer resection. There is a disproportionate excess of colorectal cancer in those born in September compared with the other months. Larger studies are required to validate this finding.

## Figures and Tables

**Table 1:** The interactions between these terms and status are demonstrated. Only the sine curve with period of 1 year and its interaction with status were significant.

Coefficients	Estimate	Error *z* value	*z* value	Pr(>|*z*|)
(Intercept)	3.99	0.027	143.642	<0.001
CRMCRC	−0.22	0.045	−5.334	<0.001
s1	0.05	0.039	1.475	0.140
CRMCRC:s1	−0.13	0.058	−2.234	0.026

CRMCRC: Colorectal Malignancy (CRC in text) CRControls.

s1 = sine term with period of 1 year.

**Table 2:** Month of birth for case and control groups. No significant differences are seen between the groups (*p* = 0.776).

	Included cases		Controls	
	*N*	%	*N*	%
January	67	6.5	98	7.7
February	84	8.6	102	8.0
March	70	6.8	139	10.9
April	77	7.5	102	8.0
May	89	8.7	138	10.8
June	89	8.7	111	8.7
July	88	8.6	113	8.8
August	100	9.8	84	6.6
September	100	9.8	89	7.0
October	83	8.1	107	8.4
November	82	8.0	96	7.5
December	90	8.8	98	7.7

Total	1019	100	1277	100
